# Clinically relevant aberrant Filip1l DNA methylation detected in a murine model of cutaneous squamous cell carcinoma

**DOI:** 10.1016/j.ebiom.2021.103383

**Published:** 2021-05-14

**Authors:** Kevin Roth, Louis Coussement, Elena V. Knatko, Maureen Higgins, Sandra Steyaert, Charlotte M. Proby, Tim de Meyer, Albena T. Dinkova-Kostova

**Affiliations:** a Jacqui Wood Cancer Centre, Division of Cellular Medicine, Ninewells Hospital and Medical School, University of Dundee, Dundee DD1 9SY, United Kingdom; b Biobix, Department of Data Analysis and Mathematical Modelling, Faculty of Bioscience Engineering, Ghent University, Coupure Links 653, 9000, Ghent, Belgium; c CRIG, Cancer Research Institute Ghent, Sint-Pietersnieuwstraat 25, 9000, Ghent, Belgium; d Department of Pharmacology and Molecular Sciences and Department of Medicine, Johns Hopkins University School of Medicine, Baltimore MD 21205, USA

**Keywords:** cSCC, DNA methylation, RRBS, FILIP1L, UVR, Ultraviolet radiation, NMSC, non-melanoma skin cancer, cSCC, cutaneous squamous cell carcinoma, ssUV mcSCC, solar simulated mouse cSCC, Tet, Ten-eleven-translocase, (ox)RRBS, (oxidative) reduced representation bisulfite sequencing, VS, ventral skin, DS, dorsal skin, iHS, independent healthy skin, CpG, 5′-Cytosinephosphate-Guanine-3′, CGI, CpG island, DMR, differentially methylated region, Filip1l, Filament A interacting protein 1-like, iso, isoform, NHK, normal human keratinocyte, AK, actinic keratosis, 5mC, 5-methylcytosine, 5hmC, 5-hydroxymethylcytosine

## Abstract

**Background:**

Cutaneous squamous cell carcinomas (cSCC) are among the most common and highly mutated human malignancies. Understanding the impact of DNA methylation in cSCC may provide avenues for new therapeutic strategies.

**Methods:**

We used reduced-representation bisulfite sequencing for DNA methylation analysis of murine cSCC. Differential methylation was assessed at the CpG level using limma. Next, we compared with human cSCC Infinium HumanMethylation BeadArray data. Genes were considered to be of major relevance when they featured at least one significantly differentially methylated CpGs (RRBS) / probes (Infinium) with at least a 30% difference between tumour vs. control in both a murine gene and its human orthologue. The human EPIC Infinium data were used to distinguish two cSCC subtypes, stem-cell-like and keratinocyte-like tumours.

**Findings:**

We found increased average methylation in mouse cSCC (by 12.8%, *p* = 0.0011) as well as in stem-cell like (by 3.1%, p=0.002), but not keratinocyte-like (0.2%, *p* = 0.98), human cSCC. Comparison of differentially methylated genes revealed striking similarities between human and mouse cSCC. Locus specific methylation changes in mouse cSCC often occurred in regions of potential regulatory function, including enhancers and promoters. A key differentially methylated region was located in a potential enhancer of the tumour suppressor gene *Filip1l* and its expression was reduced in mouse tumours. Moreover, the *FILIP1L* locus showed hypermethylation in human cSCC and lower expression in human cSCC cell lines.

**Interpretation:**

Deregulation of DNA methylation is an important feature of murine and human cSCC that likely contributes to silencing of tumour suppressor genes, as shown for *Filip1l*.

**Funding:**

British Skin Foundation, Cancer Research UK

**Research in context**

**Evidence before this study**

Cutaneous squamous cell carcinoma (cSCC) is the most common human malignancy. While most cSCCs are readily treated by surgical excision, some cSCC recur and advance to aggressive stages. Furthermore, high risk groups, such as organ transplant recipients, have a dramatically increased risk to develop aggressive and metastatic cSCCs. Currently used animal models are not able to adequately reflect the complex mutational landscape of human cSCC. We have previously developed a preclinical mouse model that is histopathologically and genetically remarkably similar to human cSCC. This model presents a unique opportunity to study cSCC biology and, more importantly, test treatment and prevention strategies to improve cSCC management.

**Added value of this study**

This study compares DNA methylation in our mouse model to data from human cSCC. We find that in our model both general DNA methylation features and differentially methylated regions are remarkably similar to human cSCC, further validating it as a good representation of human cSCC. Additionally, we identify differentially methylated regions that likely affect target gene expression and correlate with clinical features in patients.

**Implications of all the available evidence**

Because slow disease progression in cSCC complicates studies in humans, good animal models are needed to develop new prevention and intervention strategies and evaluate their benefits. Currently used models do not reflect the complex genetic and epigenetic landscape of human cSCC. The histopathological, genetic and epigenetic similarities between the solar simulated UV mouse cSCC model and human cSCC make it a valuable tool to study human cSCC. Furthermore, the differentially methylated regions identified in our study could serve as biomarkers to stratify aggressive from non-aggressive cSCC, and indicate further treatments and improve patient outcomes.

Alt-text: Unlabelled box

## Introduction

Despite the beneficial effects of ultraviolet radiation (UVR) for e.g. vitamin D production and skin nitric oxide mediated blood pressure reduction [1,2], solar UVR also poses a major health risk, and is the main etiological factor for the development of skin cancer. Environmental and lifestyle factors as well as longer life expectancy increase the cumulative lifetime UV exposure and consequently, skin cancer rates are increasing [3]. Cancers of the human skin can be classified within two general types, melanoma and non-melanoma skin cancer (NMSC). NMSC are largely comprised of keratinocyte cancers, and are further sub-divided into basal cell carcinoma and squamous cell carcinoma (cSCC) [4]. Cutaneous SCC account for at least 20% of all human skin cancers and their incidence is increasing steeply in ageing Caucasian populations [5]. While cure rates for localized cSCC are as high as 96% when appropriately diagnosed and treated, there are limited treatment options for advanced and metastatic cSCC [6]. Especially patients from high-risk groups, such as solid organ transplant recipients, face a dismal prognosis as high-risk cSCC often recur, have high rate of metastasis and treatment options are very limited [7], [8], [9].

Exposure to UVR causes DNA damage in skin cells such as keratinocytes [10,11] and leads to a very high mutation burden in cSCC with as much as 1 mutation per 30 kb of coding sequence, making cSCC the most mutated cancer [12]. The vast majority of these mutations are “UV-signature mutations”, i.e., G to A or C to T transitions, which makes G- and C-rich genes more likely to become mutated [13]. Known driver mutations in cSCC are *TP53* (early event, <65% of cSCC), *NOTCH* (early event, <75% of cSCC), *CDKN2A* (50% of cSCC, with additional epigenetic inactivation), and *TGFB1* [14], [15], [16]. Interestingly, whereas *RAS* mutations are relatively rare, commonly used animal models, such as the DMBA/TPA two-stage skin carcinogenesis model are Ras dependent [17]. To accurately model carcinogenesis and the complex genetic landscape of cSCC, we subjected SKH-1 hairless mice to chronic intermittent sub-erythemal doses of solar simulated UVR (comprised of UVA and UVB wavelengths). This mouse model produces tumours that are histopathologically and genetically very similar to human cSCC [18], [19], [20].

It is becoming increasingly clear that inactivation of tumour suppressor genes plays a major role in cSCC [21], [22], [23]. In addition to mutations, tumour suppressor genes are often silenced by DNA methylation. Furthermore, global DNA hypomethylation in cancer cells contributes to genome instability and expression of aberrant transcripts from repetitive sequences that lead to chromosomal rearrangements, mitotic recombination and aneuploidy [24], [25], [26]. Despite the global DNA hypomethylation generally found in cancer, promoters of tumour suppressor genes are often hypermethylated and thereby silenced. Our previous analysis of the mutational landscape of mouse cSCC tumours from a solar-simulated UV radiation model (hereafter termed solar-simulated UV mouse cSCC, ssUV mcSCC) identified members of the *Tet* gene family among the mutated genes [20]. *Tet* genes encode Ten-eleven-translocases that are involved in DNA demethylation [27], prompting us to investigate potential alterations in DNA methylation in cSCC. In this report, we first present a genome-scale analysis of the methylome of the ssUV mcSCC tumours, showing that DNA methylation changes often occur at regions that have genomic features commonly associated with regulatory function. We then compare DNA methylation patterns in the mouse tumours to human primary cSCCs, further validating the clinical relevance of this model.

## Methods

### Ethics

Cutaneous carcinogenesis experiments were performed in accordance with the regulations described in the UK Animals (Scientific Procedures) Act 1986. Experiments were approved by the Welfare and Ethical use of Animals Committee of the University of Dundee. Experimental design was in line with the 3Rs principles of replacement, reduction, and refinement (www.nc3rs.org.uk).

### Cutaneous carcinogenesis

Tumours and matched control skin samples were obtained from a previous study [18]. Briefly, SKH-1 hairless mice (Charles River, Germany) were bred in our facility with free access to water and food (pelleted RM1 diet from SDS Ltd., Witham, Essex, UK), on a 12 h light/ 12 h dark cycle, 35% humidity. The experimental animals were age-matched and female. Starting at 8 weeks of age, the mice were exposed twice a week for 15 weeks on Tuesdays and Fridays to solar-simulated UV radiation (comprised of 2 J/cm^2^ UVA and 90 mJ/cm^2^ UVB) delivered from UVA340 lamps (Q-Lab, Germany) in clear bedding-free cages. The radiant dose was confirmed with an external radiometer (X-96 Irradiance Meter; Daavlin, Bryan, OH) before and after each irradiation session. Excessive heating was prevented by use of an electrical fan. After the end of the irradiation schedule, the animals were monitored for a further 20 weeks, and tumours (defined as lesions >1 mm in diameter) and body weights were recorded weekly. The mice were then euthanized, individual tumours, tumour-free dorsal skin, and non-irradiated ventral skin were harvested, immediately frozen in liquid N_2_, and stored at –80 °C. Laser-capture microdissection (on Zeiss Palm Microbeam microscope, Zeiss, UK) was used to enrich for tumour cell populations and prevent contamination by infiltrating inflammatory cells. Genomic DNA was extracted from tumour cells and from matched normal skin samples. The goal of this study was to examine cutaneous squamous cell carcinomas (cSCC) in mice and the feasibility to use the mouse model as a model for human cSCCs. Therefore, we studied 7 cSCC tumour samples in mice. For each tumour sample, a matching control sample was included from an area of the skin that was not affected by the tumours. A technical replicate was included for one of the controls as well as one of the tumour samples. Additionally, two independent skin control samples were obtained from mice of the same age as the study group, living under the same conditions, but not exposed to UV radiation. All samples were subjected to (oxidative) reduced representation bisulfite sequencing ((ox)RRBS).

### Isolation of genomic DNA for oxRRBS

DNA for reduced representation bisulfite sequencing (RRBS) was obtained using the protocol described in [20]. In brief, mouse skin tumours were harvested and snap-frozen in liquid N_2_. Tumour tissue was enriched using laser capture microdissection and genomic DNA was isolated using the QIAmp DNA micro kit (Qiagen) according to the manufacturer‘s protocol. Genomic DNA was then used for oxRRBS library preparation.

### Isolation of RNA and protein for expression and protein quantification

RNA and protein were isolated from fresh frozen tissue using a combination of TRI reagent (Sigma Aldrich) and the Qiagen AllPrep DNA/RNA kit. In brief, tissue was pulverized using a mortar. Tissue powder was lysed with 500 µL RLT Plus buffer, supplemented with 1% v/v β-mercaptoethanol (β-ME). Non-soluble components were removed by centrifugation and supernatant was mixed with 1 mL of TRI reagent, incubated for 5 min at room temperature (RT) before being mixed with 200 µL of chloroform and incubated at RT for 15 min. Phase separation was achieved by centrifugation (15 min, 13000 rpm, 4°C). The colourless, aqueous phase, containing the RNA, was transferred to a new 2-mL reaction tube and incubated for 15 min at RT after addition of 500 µL 2-propanol. RNA then was bound to a Qiagen AllPrepDNA/RNA kit RNA column and isolated according to a modified version of the manufacturer's protocol. The organic phase, containing the protein, was mixed with 1 mL 2-propanol to precipitate the protein. Protein was pelleted by centrifugation (10 min, 13000 rpm, 4 °C). Protein pellets were washed twice using 1 mL wash solution) for 20 min. After the final washing step, protein pellets were either stored in washing solution at -20 °C or directly resuspended in 2x SDS buffer.

### Library preparation and sequencing

Library preparation and sequencing were performed by NXTGNT (Ghent, Belgium). Upon arrival, the samples were assessed by Quant-iT™ PicoGreen™ dsDNA Assay Kit (P7589, ThermoFisher) for quality. No aberrations were detected, and 1 µg DNA was measured for MSP1 digestion. Digestion was performed overnight for 16 h at 37 °C in a volume of 30 µl and stopped with 5 µl of 0.5 M EDTA. Subsequently, the digestion product was purified with the GeneJET PCR Purification Kit (K0701, ThermoFisher), eluted in 50µl elution buffer and quality was checked again on E-Gel™ EX Agarose Gels, 1% (G401001, ThermoFisher). NEBNext Ultra DNA Library Prep Kit for Illumina (E7370, New England Biolabs) and TrueMethyl seq kit (Feb 2015, Cambridge Epigenetix) were used for library preparations, both kits according the manufacturers recommendations. Samples were splitted into two aliquots (each 275 ng) of which one was oxidated for oxRRBS. After bisulfite conversion and subsequent clean-up reaction, polymerase chain reaction (PCR) amplification was performed. Agencourt AMPure XP Bead Clean-up 1:1 (E6260) was performed for cleanup and DNA fragment length selection. Finally, a DNA high sensitivity chip on the Bioanalyzer (Agilent technologies) and measurements of qPCR quantification according to the Illumina protocol (“qPCR quantification protocol guide”) concluded the last quality control steps.

Sequencing was performed on the NextSeq500 using 7 dark cycles on single read fragments with a length of 76 bp. A concentration of 1.8 pM was loaded with a 15% PhiX spike-in.

### Sequence read mapping and summarization

The Mouse reference genome as provided by Ensembl (GRCm38/mm10) was used for mapping of (ox)RRBS sequencing reads. Quality control and filtering of low-quality reads was performed using “Trim Galore!” (Babraham Bioinformatics). Quality control indicated no major problems, so Bismark (v.0.16.3, Babraham Bioinformatics) was used in Bowtie2-mode [28], for mapping. Seed length, mismatches and interval during multiseed alignment were set to the default values.

### Differential methylation analysis

The differential methylation analysis was performed in R (v. 3.3.1) using Bioconductor (v. 2.34.0). After summarization the data were imported using the BiSeq-package (v. 1.14.0) in R. Comparison between average methylation percentages of different states (e.g. cases vs controls) was performed using ANOVA analysis and subsequent Tukey post hoc analysis, if more than two groups were compared. Additionally, for correlation calculations, Pearson correlation was performed.

Raw counts were used to calculate methylation percentages (β-values, (1)) and subsequently M-values (2), with constant equal to 0.01. M-values were demonstrated to have superior statistical properties for Infinium HumanMethylation BeadArray data [29], but can also be applied on methylation sequencing data [30], and yield more appropriate data to be used with the R Bioconductor limma package (v. 3.30.13).

(1) $$\beta =\;\frac{\;\#\;Methylated\;reads}{\#\;Total\;reads}$$

(2) $$M=\;\log_{2}\left(\frac{\beta +C^{te}}{1-\beta \;+C^{te}}\right)\;\;\;\;\;\left(C^{te}=0.01\right)$$

The calculation of β- and M-values implies intrinsic normalization (i.e. biases are largely equal for methylated and unmethylated reads), therefore no additional normalization between samples was performed. Data were however filtered to improve quality: (i) all loci that have a minimal coverage lower than 8x were considered insufficiently informative and were removed from the dataset, (ii) a minimum of 6 methylated reads over all samples was required to be retained in the dataset for further analysis (i.e. at least some methylation should be present). Finally, after statistical analysis, the Benjamini-Hochberg procedure was used to calculate false discovery rates (FDR), and set at a threshold of 10% to indicate significance.

Additionally, the clusterSites function from the BiSeq package was used to search for agglomerations of CpG sites. A minimum of 15 CpGs in maximum 200 bp found in at least 75% of all samples are considered a potential differentially methylated region (DMR). The BiSeq package uses beta binomial regression to estimate p-values for each potential DMR [31].

### Comparison with independent data

To evaluate the relevance of the mouse model in a human context, results were compared with human cSCC Infinium HumanMethylation BeadArray data created by Rodriguez-Paredes *et al.* [32]. The data were downloaded via the ArrayExpress database (accession: EGAS00001002670) and imported using the wateRmelon package (v. 1.18.0). Probe annotation was obtained using the ChAMPdata package (v. 2.18.0) which is based on genomic coordinates for the GRCh37 reference genome. Upon importing the data, the filter function was used to filter out probes with high detection p-values. The same strategy was used for the statistical analysis as for the RRBS data, i.e. linear models of the M-values by means of limma. Due to the high sample size (*n* = 46), and thus more power, compared to the RRBS dataset (*n* = 16) a more conservative FDR cut-off (5%) was applied for the human dataset. Next to assessing differentially methylated loci, we also evaluated the identification by Rodriguez-Paredes et al. [32] of stem-cell like and keratinocyte-like tumour samples. The authors kindly provided tumour group annotation per sample.

Finally, genes were coined to be of major relevance when they featured at least one significantly differentially methylated CpGs (RRBS) / probes (Infinium) with at least a 30% difference between tumour vs. control in both a murine gene and its human orthologue.

### Clinical impact of methylation changes in human data

To assess the clinical relevance of *FILIP1L* methylation, human EPIC Infinium data published by Hervás-Marín *et al.* were downloaded from arrayExpress (https://www.ebi.ac.uk/arrayexpress/experiments/E-MTAB-8542/files/). Using the original annotation by the authors, the low-risk group corresponds to samples from initial invasive carcinoma (*n* = 10), and the high-risk group to samples from high-risk non-metastatic carcinoma or metastatic carcinoma (*n* = 8) [33]. Differential methylation analysis was performed using limma on the M-values as described above. Significantly differentially methylated CpGs within the *FILIP1L* gene were identified (*n* = 23) and compared with the clearly differentially methylated CpGs in keratinocyte-like and stem cell-like tumour samples (i.e. significant and >30% difference in mean methylation between tumour and control samples).

### Human-mouse orthologs

For establishing a human-mouse ortholog gene set, Ensembl was queried using the biomaRt package (v. 2.24.0) for R. Gene annotation of the GRCh38 assembly of the human genome contains homology information and was used in combination with the latest reference genome for mouse (assembly GRCm38), since this version is the most complete for gene symbol annotation [34]. BiomaRt gives an indication whether there is a high likelihood of two genes (one human, one murine) being orthologous or a low likelihood. In case multiple genes with a high likelihood were found, the gene with the highest degree of homology was selected. Also, in case no gene with a high likelihood was found, the gene with the highest percentage of homology was selected. In both cases, if homology percentages were equal, genes with an identical gene symbol were preferred over their fellow candidates. If the latter still resulted in redundant candidates, all were kept as candidate human-mouse orthologs.

### Visualization of genomic features in the UCSC genome browser

For visualization and integration of different data sources, results were presented as genomic tracks for the mouse GRCm38 (mm10) genome, compatible with usage in the UCSC genome browser. RRBS results were compiled as BedGraph files (https://genome.ucsc.edu/goldenpath/help/bedgraph.html). For all significant CpGs, the methylation percentage is displayed as a positive value (between 0 and 1) whereas for nonsignificant CpGs, the methylation percentage is displayed as a negative value in a different colour. Here and further in the manuscript statistical significance is defined as mentioned before (RRBS: FDR<0.10; Infinium: FDR<0.05).

Figures that illustrate genomic features at the DMRs contain the following UCSC genome browser tracks. Keratinocyte methylation data were available from He *et al.* [35] and displayed in the Keratinocyte track. Chip-Seq data for CTCF and the H3K27Ac and H3K27me3 histone marks were obtained from the Gene Expression Omnibus (GEO) using the study of Yu *et. al* [36]. The deeptools suite [37] was used to manipulate the obtained files to make them compatible for UCSC liftover tool, for adaptation of mm9 coordinates to GRCm38 (mm10) coordinates and finally back to the original bigwig format for usage in the UCSC genome browser. Finally, FANTOM5 TSS activity data were obtained to investigate whether differential methylation colocalizes with regulatory domains in the mouse genome [38].

### Clustering

Clustering was achieved by using the 10,000 most variant loci over all samples, thereby excluding noise and low informative loci. As dissimilarity measure, the Euclidian distance based on the covariance of the methylation percentages was used.

### Cell culture

Human and murine cells, primary cells and cell lines, were cultured under sterile condition in flasks and maintained at 37 °C in a humidified incubator with 5% CO_2_ (HERAcellTM incubator). Cells were ensured to be free of mycoplasma contamination by routine testing using MycoAlert® Mycoplasma Detection Kit (Lonza). Cutaneous Squamous Cell Carcinoma cells (cSCC) and normal human keratinocytes (NHK) were isolated from tumour tissue obtained from patients and from breast or abdominal normal skin of human subjects, respectively, after written and informed consent [22,39]. cSCC cell lines and NHK cells were maintained in RM+ medium with the following composition: a mixture of DMEM:Ham's F12 (3:1) (Thermo Scientific) media supplemented with 10% fetal bovine serum (FBS, Thermo Scientific), 0.4 μg/mL hydrocortisone (Sigma), 5 μg/mL insulin (Sigma), 10 ng/mL epidermal growth factor (EGF, Serotec), 5 μg/mL transferrin (Sigma), 8.4 ng/mL cholera toxin (Sigma) and 13 ng/mL liothyronine (Sigma). The mouse keratinocyte cell line Kera308 (obtained from Cell Lines Service) was maintained in DMEM, supplemented with 10% FBS. Filip1l siRNA knockdown in Kera308 cells was achieved using the reverse transfection protocol of the RNAiMax reagent kit (Thermo Fischer) according to the manufacturer's protocol. A list of siRNA assays can be found as supplementary information.

### Isoform specific qPCR

RNA was extracted and converted to cDNA using the Qiagen Omniscipt RT kit with a modified (50% reduced RT enzyme concentration) version of the manufacturer's protocol and random hexamers (Invitrogen). qPCRs were performed using a standard Taqman protocol on the QuantStudio 5 RT-PCR system (Thermo Fischer). Isoform specific Taqman probes were designed using Primer Quest online tool from Integrated DNA technologies (see https://www.idtdna.com/PrimerQuest) and sequence specificity was ensured by BLAST against the host genome and transcripts. A list of Taqman assays can be found as supplementary information.

### Immunoblotting

Proteins were separated using precast polyacrylamide (10% or 4–12%) Bis-Tris gels (Invitrogen), and MOPS (Invitrogen) as running buffer. Proteins were transferred to a 0.45 µm supported nitrocellulose membrane (Amersham – GE Healthcare Life Sciences). Transfer was completed in 1x Transfer buffer, supplied with 0.1% SDS at 100 V for 90 min using a wet transfer system. Membranes were blocked using 5% non-fat milk in 1x TBS for 1 h at room temperature. Primary antibodies were prepared in 0.1% non-fat milk in 1x TBS-T in appropriate dilutions and incubated with the membrane on a roller at 4 °C overnight. Membranes were washed 3 times for 15 min using 1x TBS-T. Secondary antibodies were diluted 1:15,000 in 0.1% non-fat milk in 1x TBS-T and incubated for 1 to 2 h at room temperature. Excess secondary antibody was washed off as described for primary antibodies. Image capture and analysis were done using the Odyssey® CLx image system and Image Studio software (LI-COR). A list of used antibodies can be found as supplementary information.

### Role of funding source

The funding bodies had no influence on study design, data collection, data analyses, interpretation, or writing of report.

## Results

### DNA methylation discriminates between murine tumours and normal skin

We analysed DNA methylation and hydroxymethylation changes in cSCC tumours obtained from our previously developed ssUV mcSCC model [18,20]. Six laser-capture micro-dissected tumour samples, six matched controls (non-irradiated ventral skin from the same animal), and two independent healthy skin samples (dorsal skin from age-matched animals) were subjected to oxidative reduced representation bisulfite sequencing (oxRRBS). Two technical replicates (one tumour, one matched control) were included to control for data quality. The analysis of the levels of 5hmC showed that hydroxymethylation was extremely low and therefore not distinguishable from noise (see Supplementary Figure 1). We therefore used the conventional RRBS data throughout the remainder of this study.

**Fig. 1:**
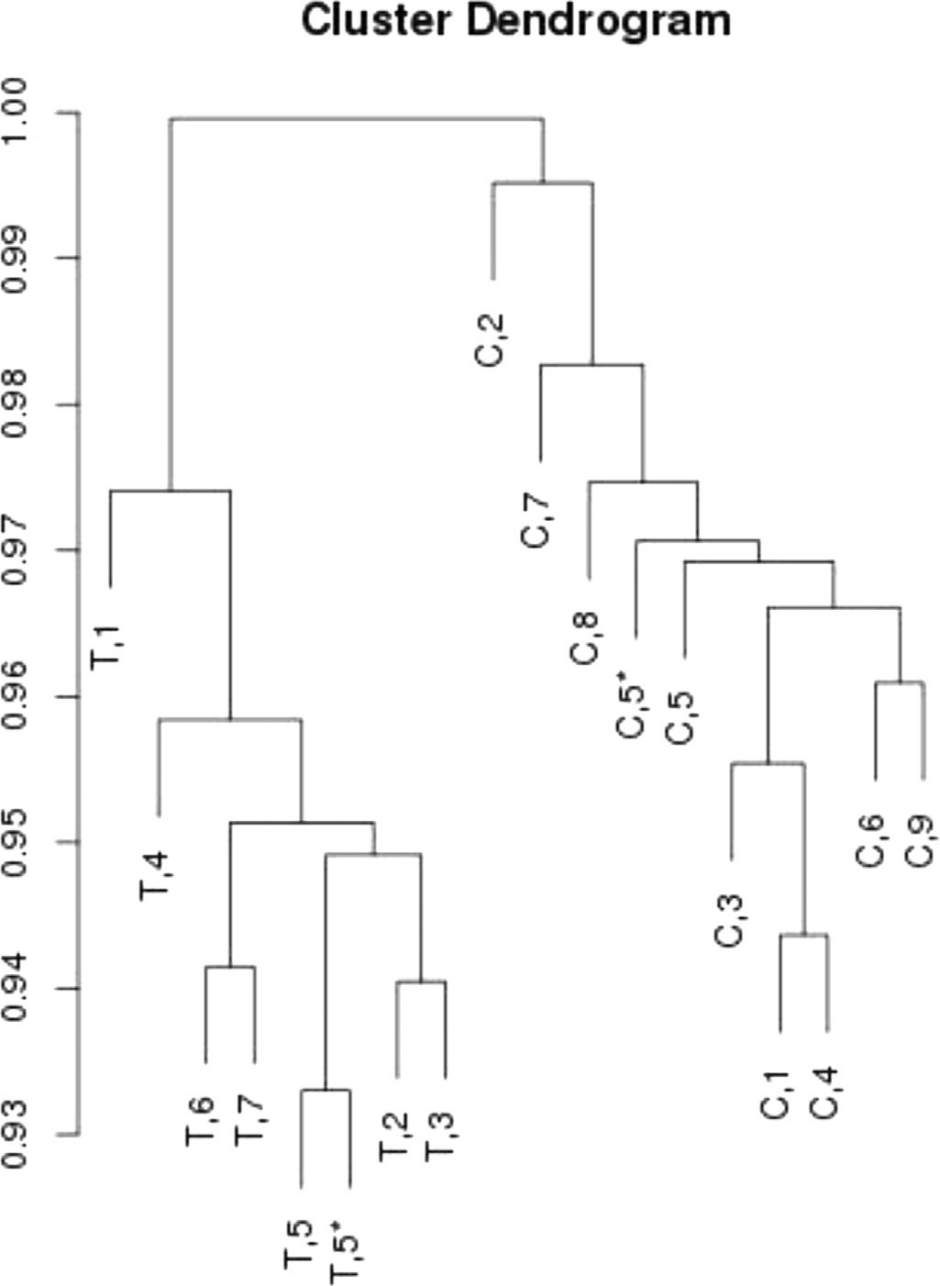
Cluster analysis indicates larger difference between tumours and controls than between individuals. T and C indicate tumour and control samples respectively, the number indicates the animal of origin and finally, technical replicates are indicated by an “*”. C,8 and C,9 represent the two independent healthy skin samples which cluster with the other control samples, indicating that methylation does not discriminate normal ventral from dorsal skin. Tumour and control samples cluster separately, indicating profound methylation changes between the two groups. Cluster analysis was performed using the 10,000 most variant loci.

Based on clustering of the samples (Fig. 1), changes due to tumour proliferation are more evident than differences between individuals, i.e. the highly disruptive character of skin cancer is also observable at the DNA methylation level. Furthermore, the cluster analysis shows that technical replicates cluster together, indicating good quality of the data. Technical replicates were therefore merged and treated as a single sample in further analyses.

### Average DNA methylation is higher in tumours compared to controls

In most cancers, large stretches of intergenic DNA become hypomethylated, while DNA methylation at CpG islands become either hypo- or hypermethylated leading to a global decrease in DNA methylation levels [40,41].

**Fig. 2:**
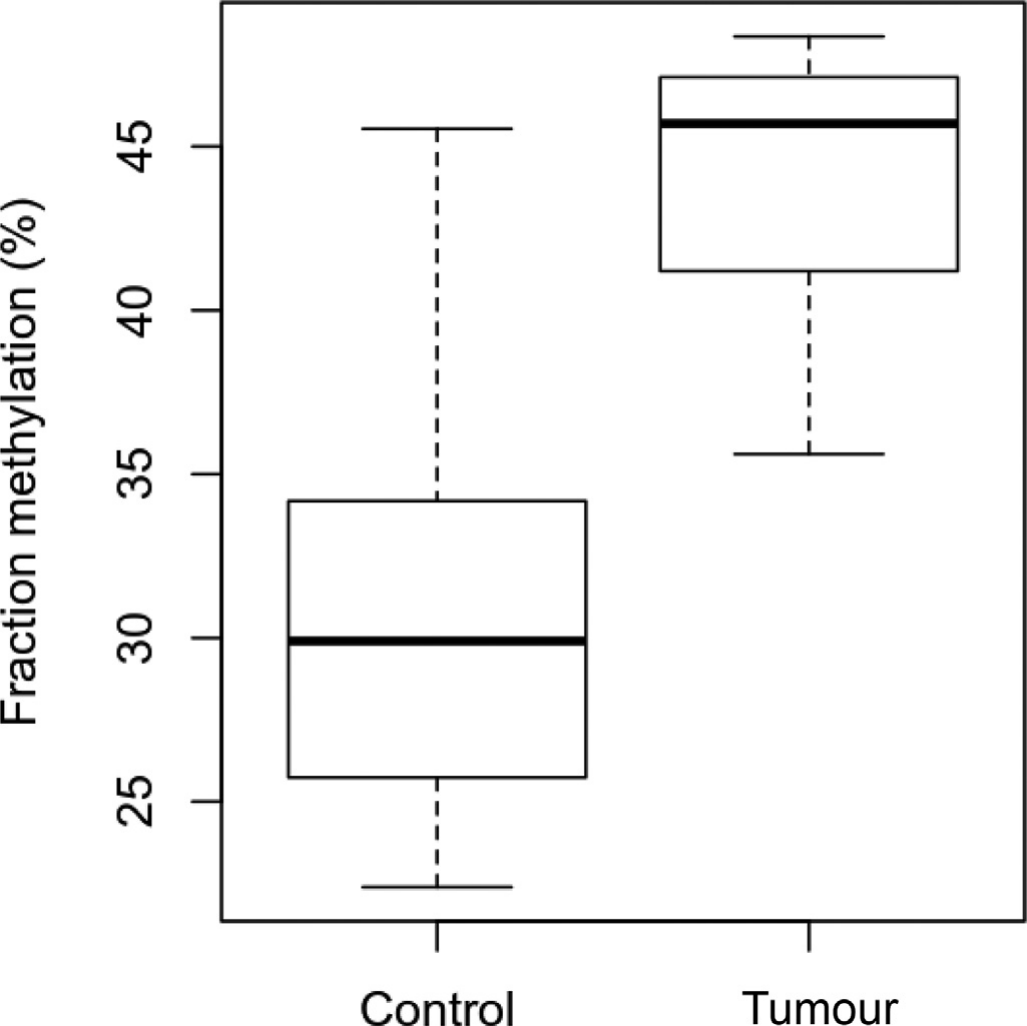
Average methylation is significantly higher in mouse ssUV cSCC compared to matched controls. Note that average methylation levels are based on the results from 86,508 sufficiently covered CpGs in the RRBS data (< 0.4% of all mouse CpGs), which are not necessarily representative for the full genome.

We detected a 12.8% (*p* = 0.0011; ANOVA with Tukey post hoc) increase in average DNA methylation in murine tumour samples (Fig. 2). However, note that our RRBS strategy only analysed a sub-fraction (86,508 CpGs) of the approximately 22 million CpGs present in the murine genome (GRCm38/mm10 assembly), typically in CpG dense regions, which may confound the extrapolation of this conclusion to the full genome.

### Differentially methylated regions co-localize with regulatory elements

Differential methylation was assessed at the CpG level using limma, resulting in 640 differentially methylated CpGs in 318 unique genes (see Supplementary Table 1). However, here we particularly focus on differentially methylated regions (DMR), as these are deemed to have more impact than individual CpGs. Using the BiSeq package, 78 significantly differentially methylated regions (DMRs) were found in tumours versus controls (76 hypermethylated, 2 hypomethylated). The top 20 DMRs are depicted in Table 1 (for full DMR list see Supplementary Table 2). Median differences in methylation ranged from -13% to 40% (positive values indicate increased methylation in tumours). The most striking result was a DMR on chromosome 16 (position 57,391,482-57,391,657) that featured the largest difference in methylation (40% increased median methylation in tumours). The region overlaps with two genes on opposite strands, *Filip1l* and *Cmss1*.

**Table 1:** Top 20 DMRs with the highest difference in methylation between controls and tumours. Of the top 20 DMRs, 19 are hypermethylated and only 1 is hypomethylated in tumours compared to controls. The most differentially methylated DMR is located on chromosome 16 and overlaps with two genes encoded on opposite strands: Filip1l and Cmss1.

									Median methylation		
Gene	**Type**	**Chromosome**	**Start**	**End**	**Width**	**# CpGs**	**Median p**	**FDR**	**Control**	**Tumours**	**difference**
*Filip1l*	genic	16	57391482	57391657	176	38	0,0001421	0,0090997	34,23%	76,02%	39,87%
	intergenic	3	5860619	5860741	123	30	0,0013334	0,0613401	24,67%	57,86%	33,16%
	intergenic	12	112802696	112802752	57	9	0,0018667	0,0709347	13,80%	38,92%	25,12%
	intergenic	GL456378.1	27849	27988	140	18	0,0002308	0,0140796	12,44%	38,05%	24,52%
*Rbmxl2*	genic	7	107210000	107210046	47	24	0,0033155	0,0943661	38,22%	59,05%	20,82%
*Sp8*	genic	12	118849494	118849660	167	23	0,0010454	0,0491377	7,77%	25,01%	17,23%
*Adra2c*	genic	5	35280820	35280957	138	21	0,0032540	0,0943661	13,46%	29,93%	16,01%
*Hapln4*	genic	8	70088183	70088238	56	9	0,0000565	0,0039047	4,14%	19,43%	15,29%
*Foxd3, Gm23366, Gm12688*	promoter	4	99656874	99656946	73	13	0,0014836	0,0667642	6,11%	20,53%	14,41%
*Gm42418*	promoter	17	39845285	39846122	838	123	0,0004844	0,0256759	14,54%	31,16%	13,47%
*Cdk8*	genic	5	146261012	146261147	136	24	0,0000006	0,0000511	8,42%	21,89%	13,47%
	intergenic	11	109011699	109011975	277	37	0,0000031	0,0002340	17,71%	31,59%	13,44%
*C1ql2*	promoter	1	120341073	120341333	261	15	0,0082421	0,1127978	8,89%	22,08%	13,19%
*Gm42418, AY036118*	promoter	17	39847522	39847638	117	2	0,0245761	0,1127978	18,49%	31,49%	13,00%
*Rims1*	genic	1	22533835	22533906	72	10	0,0016663	0,0678245	5,20%	18,05%	12,86%
	intergenic	12	57538740	57538787	48	8	0,0070264	0,1127978	6,54%	18,45%	11,87%
	promoter	17	39846535	39846769	235	14	0,0052170	0,1103093	14,54%	34,12%	11,68%
*Gabra5*	promoter	7	57509682	57509946	265	17	0,0027055	0,0865786	4,65%	14,80%	10,86%
*Gm26917*	genic	17	39844548	39844900	353	68	0,0055337	0,1103093	10,53%	20,33%	9,73%
	intergenic	2	98666262	98666273	12	4	0,0093025	0,1127978	81,05%	67,73%	-13,34%

**Fig. 3:**
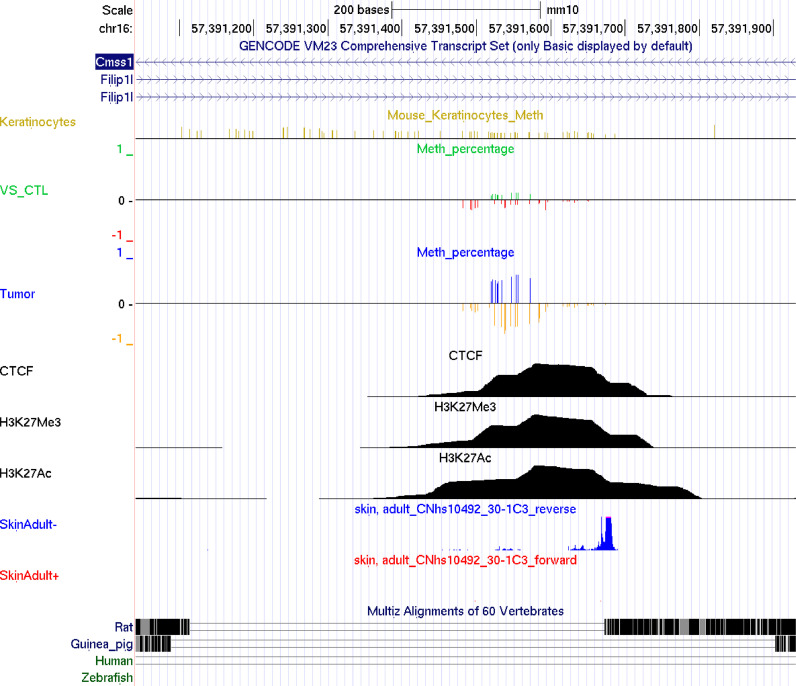
Methylation and genomic features of the DMR at the Filip1l locus. The Filip1l DMR is the most differentially methylated region we detected. The Keratinocyte track shows all CpG positions in the mouse genome. Note that RRBS does not cover all CpGs and therefore, only data for covered CpGs is displayed in the following tracks. Methylation levels in ventral skin controls and tumours are visualized in Control and Tumour tracks. Genomic features of at the Filip1l locus suggest that the region is likely an enhancer. The region is occupied by CTCF, as well as the mutually exclusive histone marks H3K27me3 (repressive) and H3K27Ac (activating). Furthermore, transcriptional activity at the DMR, highlighted in the FANTOM5 TSS activity track (“SkinAdult”) may resemble enhancer RNAs. We hypothesise that DNA methylation status of the region influences the enhancers activity.

Next, we investigated genomic features of these most discriminating DMRs. As shown in Fig. 3, the DMR covering intronic regions of *Cmss1* and *Filip1l* is hypermethylated in tumours. ChIP-Seq data from mouse skin [36] show occupancy of the region by CTCF as well as the H3K27me3 and H3K27Ac histone marks. While CTCF can block communication between enhancers and promoters [42], H3K27me3 is a mark found at repressed enhancers and has a mutually exclusive relationship with H3K27Ac, a mark of active enhancers [43,44]. We therefore hypothesize that differential methylation at the region affects enhancer activity [45]. The region in which the DMR resides is an intron for which transcriptional activity is observed (see FANTOM5 TSS activity tracks SkinAdult - and +), in line with enhancer RNA expression, a common occurrence at enhancers [46]. In summary, the genomic features of the region suggest that it is likely an enhancer, potentially controlling expression of the nearby gene *Filip1l.* As *Filip1l* encodes a tumour suppressor [47], [48], [49], we further focus on this locus for subsequent analyses.

We found genomic features similar to those found at the *Filip1l* locus at multiple other DMRs (see supplementary information). As tumour suppressor gene inactivation is an important feature of cSCC [21], [22], [23], differential methylation at regions of potential regulatory relevance is likely an important process of tumour suppressor silencing in cSCC as indicated by the *Filip1l* locus.

### DNA methylation is similar in ssUV mcSCC and human cSCC

Data from previous studies suggest that the ssUV mcSCC model is a good representation of human cSCC in terms of genetics and histopathology [19,20]. To elucidate if DNA methylation in the ssUV mcSCC model represents human cSCC, we used data from Rodriguez-Paredes *et al*. [32], who analysed DNA methylation patterns of 34 human skin samples (including 18 cSCC and 16 human actinic keratosis (AK), the precursor lesion of cSCC) using Illumina's Infinium EPIC 850k arrays. Independent of the precursor status, they found two subclasses of AK and cSCC, termed keratinocyte- and stem cell-like, featuring major DNA methylation differences at keratin gene clusters.

**Fig. 4:**
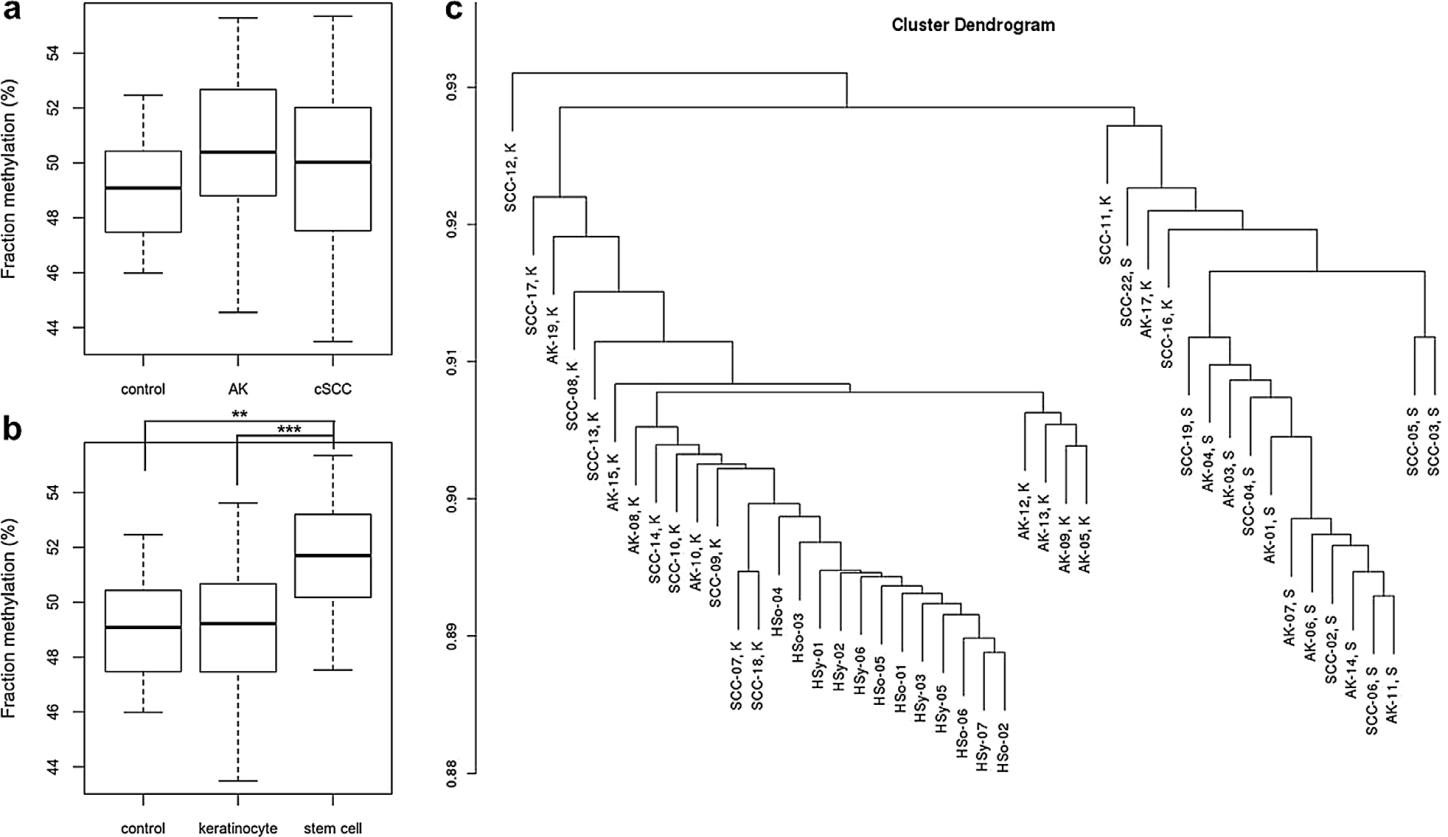
a: Average methylation of human skin samples (control), AK and cSCC. No difference in average methylation between the groups was detected. b: Average methylation of control samples and AK and cSCC samples, identified as keratinocyte like of stem cell like. Average methylation is significantly higher in stem cell like samples compared to both control and keratinocyte like samples. Significance is indicated by asterisks (ANOVA with Tukey post-hoc correction, * *p* < 0.05, ** *p* < 0.01, *** *p* < 0.001). c: Cluster plot: HSo and HSy are control samples, cSCC and AK are tumour samples; for the latter K and S indicate whether the sample is respectively a keratinocyte like or stem cell like cancer sample. With the exception of three keratinocyte-like samples, average methylation separates controls as well as keratinocyte-like and stem cell-like AK and cSCC samples.

Since average methylation was significantly higher in ssUV mcSCC tumours compared to controls in our dataset, we evaluated whether this was also the case in human cSCC. Using the data from Rodriguez-Paredes *et al.* [32], there were no overall differences between controls, AK and cSCC (Fig. 4a). However, average DNA methylation was significantly higher in the stem cell-like class compared to controls (by 3%, p = 0.01; ANOVA with Tukey post hoc; Fig. 4b), but was not increased in the keratinocyte class (0.2%, *p* = 0.98; ANOVA with Tukey post hoc). Moreover, cluster analysis of the human methylation data indicated that the large majority of stem cell-like samples clusters separately from controls and keratinocyte-like samples (Fig. 4c), supporting major general methylation differences between both AK and cSCC subclasses. Together, these findings suggest that an increase in DNA methylation levels correlates with a cSCC stem cell-type and aggressiveness and that the ssUV mcSCC model may represent a more aggressive cSCC type.

As DMRs cannot be readily compared between mouse and human, as well as between different platforms (RRBS versus Infinium array), we focused on genes containing differentially methylated CpGs in both species. Since our results support the presence of two distinct subtypes, i.e. keratinocyte and stem-cell like, analyses are performed for both subtypes separately. We found 376,297 (= 43.8%) and 362,447 (= 42.2%) significantly differentially methylated CpG dinucleotides in 27,187 (= 87.6%) and 25,877 (= 83.3%) unique genes respectively for keratinocyte and stem-cell like tumour samples in the human Infinium data (Supplementary Tables 3 and 4). We then selected genes that had at least one differentially methylated CpG in the murine data, resulting in a set of 265 genes with their human ortholog. Remarkably, these genes were more likely to be significantly differentially methylated in human tumours: 93.5% and 90.6% genes contained at least one differentially methylated CpG, compared to the respective baselines of 87.6% and 83.3% for keratinocyte and stem-cell like tumour samples respectively (chi-squared test, Bonferroni adjusted *P*-values = 0.0083 and 0.0044). In line with the hypermethylation observed in stem-cell like and murine cases, the direction of the methylation difference was significantly more concordant with stem-cell than with keratinocyte like samples (82.5% and 65.7%; chi-squared test, *P*-value = 3.8E-5).

Subsequently, we focussed on genes most likely playing a major role in cSCC, i.e. showing at least one clearly differentially methylated CpG/probe (difference in methylation of at least 30%) for both human and murine data. For both keratinocyte and stem-cell like tumour samples, we found the *Filip1l* and *Cmss1* genes to be differentially methylated in both, human and mouse cSCC. *Filip1l/Cmss1* are located at the most differentially methylated region and their hypermethylation in human cSCC highlights the importance of the genes. Additional genes identified include *Tspan9, Hoxd3/Hoxd4, Abr, Gpc6, Gal3st3, Sept9* and *Lbra/Mab21l1.* This analysis also yielded some subtype specific results: *Tcfl5, Rgma, Galnt13, Slc2a10* and *R3hdm4* for stem-cell like cSCCs, but no additional genes for keratinocyte-like. When focusing solely on promoter regions, similar trends were observed, yet typically less outspoken and with fewer overlap between genes, largely due to the fact that many relevant promoters were not covered by RRBS. Overall, the remarkable similarities in the methylomes of human and mouse ssUV cSCC show that the tumours that form in the ssUV mcSCC model, in addition to their histopathology and genetics, are also similar to human cSCC on the DNA methylation level, thus highlighting the clinical relevance of this model.

### Differential methylation at the *Filip1l* locus affects Filip1l expression

**Fig. 5:**
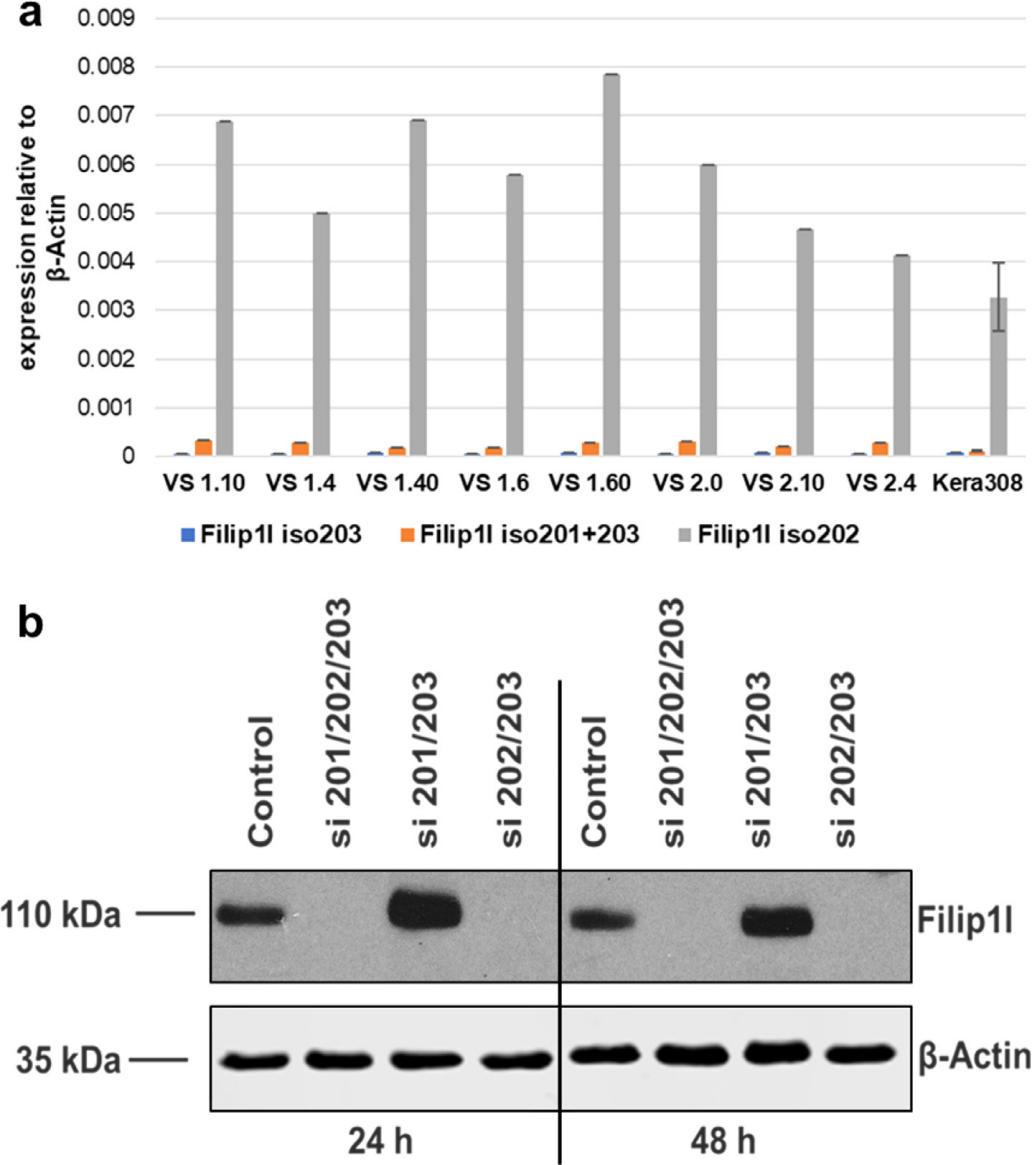
a: Isoform-specific qPCR for Filip1l in mouse ventral skin (VS, n = 8) and the mouse keratinocyte cell line Kera308. Results for Kera308 cells represent the mean of three technical replicates, error bars represent standard deviation. VS: Ventral skin, numbers indicate animal designation. The expression of isoforms 201 and 203 is minimal compared to the expression of isoform 202. We therefore conclude that isoform 202 is the main expressed isoform in mouse ventral skin. b: Protein levels of Filip1l after siRNA treatment at 24- and 48-hours post-transfection. The used siRNAs target two isoforms, either 201 and 203, or 202 and 203. si 201/203 and si 202/203 experiments use two siRNAs each, si 201/202/203 use the combination of 4 siRNAs. The combination of siRNAs targeting all Filip1l isoforms completely abolishes Filip1l protein expression, while siRNAs targeting isoforms 201 and 203 do not have an effect on Filip1l protein levels. Treatment with siRNAs targeting isoforms 202 and 203 also suppress Filip1l expression. This confirms the finding from the mRNA analysis, that Filip1l isoform 202 is the main expressed isoform in Kera308 cells.

As described earlier, we detected a DMR in an intronic region of the *Filip1l* gene that shows genomic features commonly associated with enhancers. Since there is no published information about the role of Filip1l in the skin, we subsequently characterized the isoform composition and expression of Filip1l in the mouse skin. By utilizing a combination of isoform specific qPCR probes and isoform specific siRNAs, we determined that Filip1l isoform 202 (Ensemble annotation) is the only expressed isoform in mouse skin and the mouse keratinocyte cell line Kera308 (Fig. 5). Interestingly, this isoform shows high similarities to the human FILIP1L isoform 203 (91% identity as determined by protein blast, Ensemble annotation), which has been linked to aggressiveness and metastatic potential in ovarian, pancreatic and prostate cancer, and is an independent prognostic marker in ovarian cancer [47,[50], [51], [52], [53]].

**Fig. 6:**
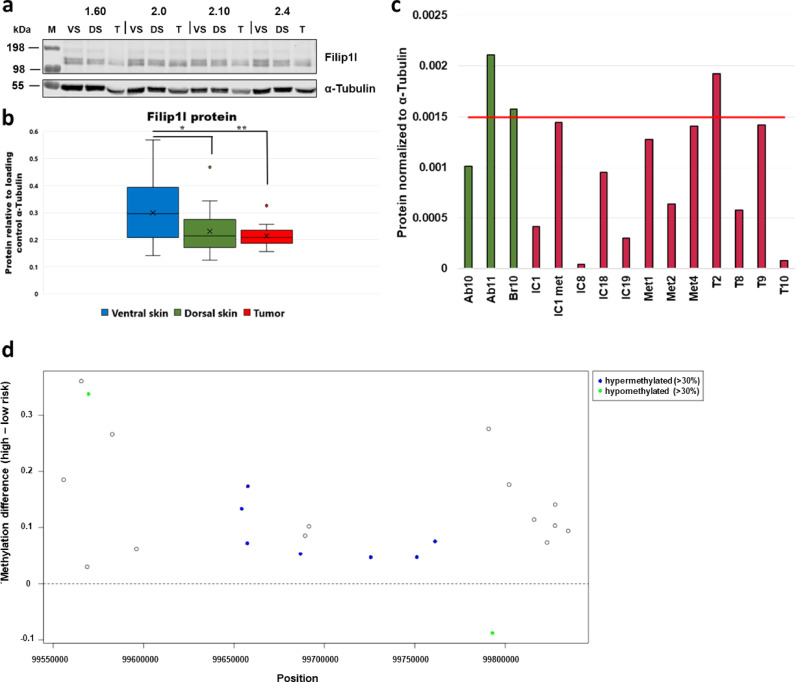
a,b: Filip1l protein levels in VS, DS and tumours of SKH-1 hairless mice that had been chronically exposed to UV radiation. a: Representative immunoblot for 4 out of 18 animals. The numbers on the top indicate individual animals. Filip1l is detected as a band at 110 kDa, consistent with its molecular weight of 98 kDa. Filip1l appears as a double band in murine skin. b: Quantification of Filip1l protein levels in all 18 animals. Filip1l protein levels are significantly reduced in DS compared to VS and further reduced in tumours compared to VS. Significance is indicated by asterisks (one-tailed paired t-test; * *p* < 0.005, **p < 0.01, ***p < 0.001). c: FILIP1L protein levels in 3 primary cultures of normal human keratinocytes (NHK, green) and 12 human cSCC cell lines (red). The red line indicates the mean FILIP1L protein levels in NHKs. FILIP1L protein levels are reduced in 7/12 cSCC cell lines. d: Methylation difference between high- and low-risk cSCC. Most CpGs at the FILIP1L locus are hypermethylated in human high-risk cSCC compared to low-risk cSCC (Hervás-Marín data [33]). Furthermore, 7/23 of these CpGs were also hypermethylated (>30%, indicated in blue) in stem-cell like compared to keratinocyte like human cSCC Rodriguez-Paredes data [32]) and 2 CpGs were hypomethylated (<30%, indicated in green).

In order to determine expression of the Filip1l protein in mouse cSCC tumours and control skin, immunoblotting was used. A total of 54 samples from 18 animals (three samples per animal: ventral skin (VS, matched control, non-irradiated), dorsal skin (DS, chronically irradiated but not tumourous) and tumour (numbers indicate animal designations) were analysed as shown in Fig. 6a,b. Using fluorescence detection immunoblotting (LI-COR), we quantified the Filip1l protein levels in all samples using α-tubulin as loading control. The Filip1l protein levels were significantly reduced in chronically-irradiated DS compared to non-irradiated VS (paired t-test, *p* = 0.03). Furthermore, in murine cSCC tumours, the Filip1l protein levels were further reduced compared to VS (paired *t*-test, *p* = 0.0026). Additionally, Fig. 6c shows the quantification of FILIP1L protein levels in a panel of 12 human cSCC cell lines [39]. Compared to the mean of three cultures of primary human keratinocytes (NHK), FILIP1L protein levels were increased in one cSCC cell line (T2), similar to NHK in 4/12 cSCC cell lines (IC1 met, Met1, Met4 and T9) and lower (i.e. below 2/3 of NHK means) than NHKs in 7/12 cSCC cell lines (IC1, IC8, IC18, IC19, Met2, T8 and T10).

Having confirmed that differential *FILIP1L* methylation and expression is also present in human cSCC, we evaluated its clinical relevance in humans using Infininium MethylationEPIC data provided by Hervás-Marín *et al.* for 10 low (initial invasive carcinoma) and 8 high-risk (high-risk non-metastatic carcinoma and metastatic carcinoma) samples [33]. Note that a high-risk assessment is associated with a higher frequency of local recurrence, lymph node metastasis and significant morbidity and mortality and thus helps identifying patients with poor outcomes. For the *FiILIP1L* gene, 23 CpG were differentially methylated (FDR < 0.05) between high- and low-risk samples. Of these, 9 had also been found to be clearly (>30%) and significantly differentially methylated in human stem cell-like samples (and less clearly, but for the majority still significantly, in keratinocyte samples) *vs.* controls. The direction of differential methylation for high-risk *vs.* low-risk was the same as for tumour *vs.* controls for 8 out of 9 probes (*P* < 0.05, binomial test), the non-consistent CpG being situated in the 3’ UTR; see Fig. 6d). This finding demonstrates that *Filip1l* methylation differences in the murine model are not only also present in human cases vs. controls, but are also clinically relevant.

## Discussion

The biology of cSCC, a type of tumour with an enormous mutational burden and no clear drivers of disease progression, is incompletely understood. In this study, we show that in contrast to most cancers which are characterized by global DNA hypomethylation, hypermethylation defines the methylome of cSCC. Our findings are supported by independent reports showing a pattern of DNA hypermethylation in high-risk non-metastatic and metastatic human cSCC [33]. Using the Infinium MethylationEPIC BeadChips 850K array (Illumina Inc. USA), these researchers found that, in addition to CpG islands, the increase in DNA methylation also occurs at CpG shelves, shores and open sea, next to CpG islands, suggesting that the observed hypermethylation is genome-wide. Moreover, considering that in cSCC most mutations do not occur within oncogenes, but within tumour suppressor genes [8,54,55], our findings indicate that in addition to loss-of-function mutations, changes in DNA methylation have a role in silencing tumour suppressor genes in cSCC.

Together, these findings suggest that DNA methylation changes are significant contributors to tumour progression in cSCC and offer potential therapeutic approaches with small molecules that have the ability to alter the DNA methylome. One example is the isothiocyanate sulforaphane, a compound that has shown protection against UV radiation-induced skin damage in both mice and humans [56], and currently is in clinical trials for several disease indications [57]. Importantly, sulforaphane has been shown to attenuate a number of UVR-induced DMRs, e.g. within *Notch1* and *Smad6*, in the mouse skin and reduce the incidence and multiplicity of skin cancer [58], in agreement with our early observations using broccoli extracts as delivery vehicles for sulforaphane [59,60].

One limitation of our study is the lack of available data for clinical outcomes in patients with cSCC. These tumours are visible and surgically excised shortly after diagnosis in order to prevent the possibility for metastatic spread, which has precluded us from correlating our findings with clinical outcomes. Another limitation of our study is that the precise function of FILIP1L in skin and cSCC remains unknown. Further investigations are needed to determine the importance of the observed downregulation of FILIP1L for the development of cSCC, and the underlying molecular mechanism(s). The majority of the available functional studies on FILIP1L have focussed on its role in modulating the WNT/β-catenin signalling pathway. FILIP1L has been shown to facilitate the destruction of β-catenin and therefore supress canonical WNT/β-catenin signalling [53,61,62], but the exact mechanism remains unknown. In this context, it is noteworthy that the study by Hervás-Marín *et al.* [33] identified the WNT/β-catenin signalling pathway as one of the potential pathways that are dysregulated in the transition of human cSCC to a high-risk stage. Though alternative RRBS data analysis methods such as the differential methylation analysis package (DMAP) [63] could have further improved the robustness of our results, it should be noted that we used two different methods, i.e. BiSeq (at DMR level) and limma (at CpG level), leading in both cases to the same conclusion for FILIP1L, which we further validated by evaluating its methylation in human cSCC tumours and assessing its protein levels in mouse skin and skin tumours, and in human keratinocytes and cSCC cell lines.

Interestingly, a recent RNA-seq analysis in BT20 breast cancer cells (which carry an activating PIK3CA mutation) identified *FILIP1L* as the most upregulated gene following treatment with vitamin C, and further implicated KDM5-mediated H3K4 demethylation in the mechanism of *FILIP1L* upregulation [64] suggesting that in addition to DNA methylation, histone H3K4 methylation has a role in the regulation of *FILIP1L* expression. It is also noteworthy that in ovarian, prostate and pancreatic cancer, *FILIP1L* expression is silenced by DNA hypermethylation; this has been linked to aggressiveness and metastatic potential, and is even considered an independent prognostic marker in ovarian cancer [47,[50], [51], [52], [53]]. It is therefore tempting to speculate that the hypermethylation and lower expression of *FILIP1L* in cSCC has a similar role. Further research is needed to test this possibility. Nonetheless, our study clearly shows that DNA methylation at the *FILIP1L* promoter is increased in high-risk human cSCC as compared to low-risk cSCC. Methylation biomarkers discriminating low- from high-risk cSCC, such as the *FILIP1L* locus, may identify patients benefitting from a more aggressive treatment regime and could therefore be a valuable tool to lower the number of recurring cSCC.

## Contributors

KR wrote the manuscript, did exploration of the DNA methylation data and all experimental work concerning Filip1l. LC performed the DNA methylation analysis and contributed equally to writing the manuscript. EK and MH performed the cutaneous carcinogenesis experiments. SS performed mapping of DNA methylation data. CP isolated, established and provided the cSCC cell line panel. TdM and ADK obtained funding, supervised the study, and participated in writing of the manuscript. All authors read and approved the final version of the manuscript.

## Declaration of Competing Interests

The authors have nothing to disclose.

## Data sharing

Data can be accessed via the Gene Expression Omnibus (GEO, GSE151657).

## Appendix Supplementary materials

Image, application 1

Image, application 2

Image, application 3

Image, application 4

Image, application 5

Image, application 6

Image, application 7

Image, application 8
