# Supplementary material for: Clinically relevant aberrant Filip1l DNA methylation detected in a murine model of cutaneous squamous cell carcinoma
Source: eBioMedicine. 2021 May 14;67:103383. doi: 10.1016/j.ebiom.2021.103383 (PMC8138604; doi:10.1016/j.ebiom.2021.103383)
Supplement: Supplementary file 1 [file mmc1.docx]

**Supplementary information legends**

**Supplementary Figure 1: Histogram of frequency of percentage of hydroxymethylation.** The vast majority of analysed loci show no hydroxymethylation (see x-axis: 0). The overall distribution is centred around zero, indicating that hydroxymethylation is not distinguishable from noise, even for the few loci exhibiting higher 5hmC content.

**Supplementary Figure 2:** **Methylation and genomic features of DMR02.** The Keratinocyte track shows all CpG positions in the mouse genome. Note that RRBS does not cover all CpGs and therefore, only data for covered CpGs is displayed in the following tracks. Methylation levels in ventral skin controls and tumors are visualized in Control and Tumor tracks. Genomic features of at the locus suggest that the region is likely an enhancer. The region is occupied by CTCF, as well as the mutually exclusive histone marks H3K27me3 (repressive) and H3K27Ac (activating). Furthermore, transcriptional activity at the DMR may, highlighted in the FANTOM5 TSS activity track (“SkinAdult”) resemble enhancer RNAs. We hypothesise that DNA methylation status of the region influences the enhancers activity.

**Supplementary Figure 3:** **Methylation and genomic features of DMR03.** The Keratinocyte track shows all CpG positions in the mouse genome. Note that RRBS does not cover all CpGs and therefore, only data for covered CpGs is displayed in the following tracks. Methylation levels in ventral skin controls and tumors are visualized in Control and Tumor tracks. Genomic features of at the locus suggest that the region is likely an enhancer. The region is occupied by CTCF, as well as the mutually exclusive histone marks H3K27me3 (repressive) and H3K27Ac (activating). Furthermore, transcriptional activity at the DMR may, highlighted in the FANTOM5 TSS activity track (“SkinAdult”) resemble enhancer RNAs. We hypothesise that DNA methylation status of the region influences the enhancers activity.

**Supplementary Figure 4:** **Methylation and genomic features of DMR04.** The Keratinocyte track shows all CpG positions in the mouse genome. Note that RRBS does not cover all CpGs and therefore, only data for covered CpGs is displayed in the following tracks. Methylation levels in ventral skin controls and tumors are visualized in Control and Tumor tracks. Genomic features of at the locus suggest that the region is likely an enhancer. The region is occupied by CTCF, as well as the mutually exclusive histone marks H3K27me3 (repressive) and H3K27Ac (activating). Furthermore, transcriptional activity at the DMR may, highlighted in the FANTOM5 TSS activity track (“SkinAdult”) resemble enhancer RNAs. We hypothesise that DNA methylation status of the region influences the enhancers activity.

**Supplementary Figure 5:** **Methylation and genomic features of DMR05. DMR05 one of the two DMRs that is hypomethylated in Tumors.** The Keratinocyte track shows all CpG positions in the mouse genome. Note that RRBS does not cover all CpGs and therefore, only data for covered CpGs is displayed in the following tracks. Methylation levels in ventral skin controls and tumors are visualized in Control and Tumor tracks. Genomic features of at the locus suggest that the region is likely an enhancer. The region is occupied by CTCF, as well as the mutually exclusive histone marks H3K27me3 (repressive) and H3K27Ac (activating). Furthermore, transcriptional activity at the DMR may, highlighted in the FANTOM5 TSS activity track (“SkinAdult”) resemble enhancer RNAs. We hypothesise that DNA methylation status of the region influences the enhancers activity.

**Supplementary Table 1: Significantly differentially methylated individual CpGs.**

**Supplementary Table 2: Detected significant DMRs.** Of the 71 detected DMRs, 69 are hypermethylated and only 2 is hypomethylated in tumors compared to controls.

**Supplementary Table 3: DNA methylation in keratinocyte-like human AK and cSCC**

**Supplementary Table 4: DNA methylation in stem cell-like human AK and cSCC**

**Supplementary Table 5: Custom designed siRNAs and Taqman probes as well as Antibodies used.**
