# Supplementary material for: Clinically relevant aberrant Filip1l DNA methylation detected in a murine model of cutaneous squamous cell carcinoma
Source: eBioMedicine. 2021 May 14;67:103383. doi: 10.1016/j.ebiom.2021.103383 (PMC8138604; doi:10.1016/j.ebiom.2021.103383)
Supplement: Supplementary file 8 [file mmc8.pptx]

## Slide 1
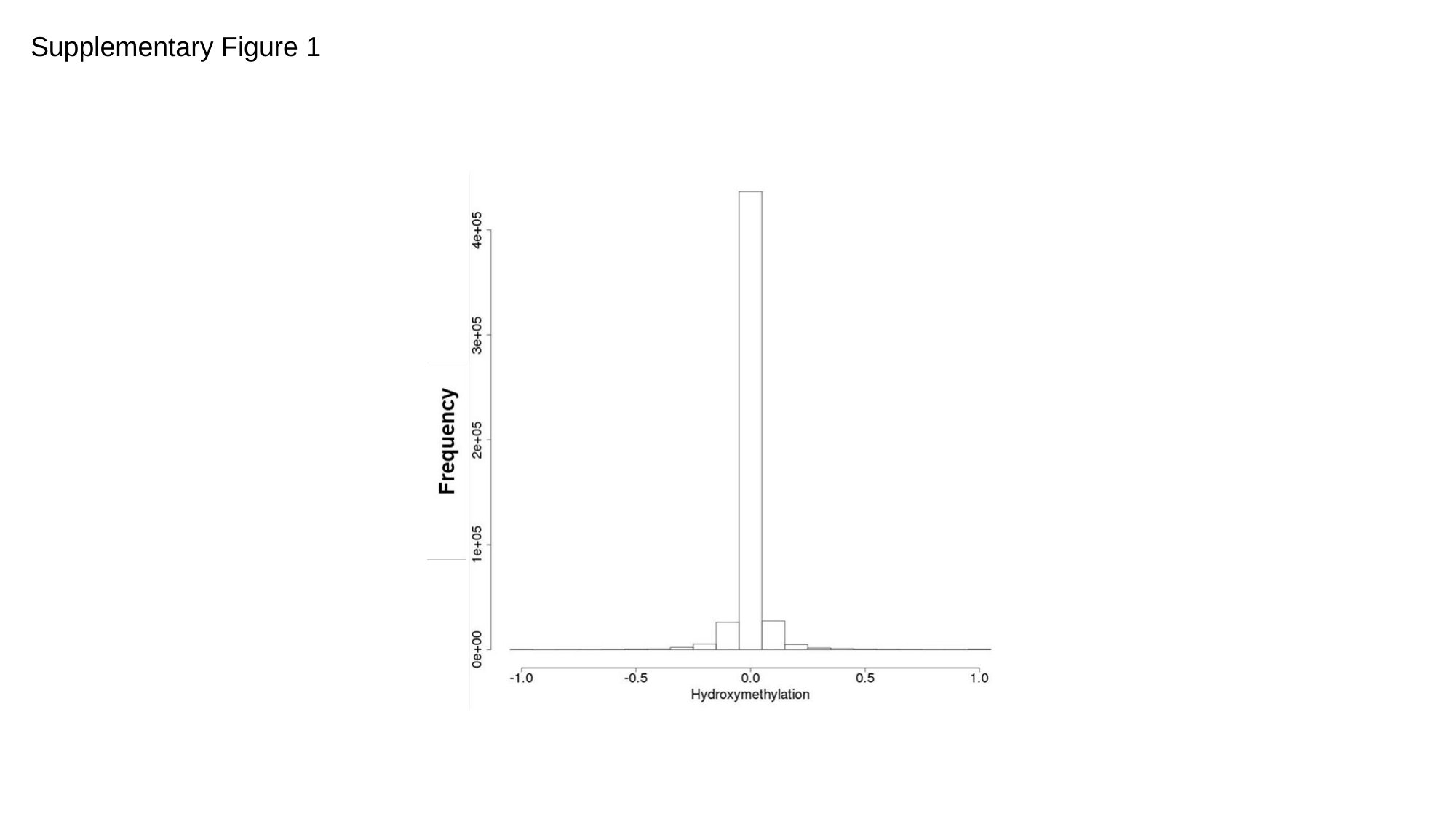

Supplementary Figure 1

## Slide 2
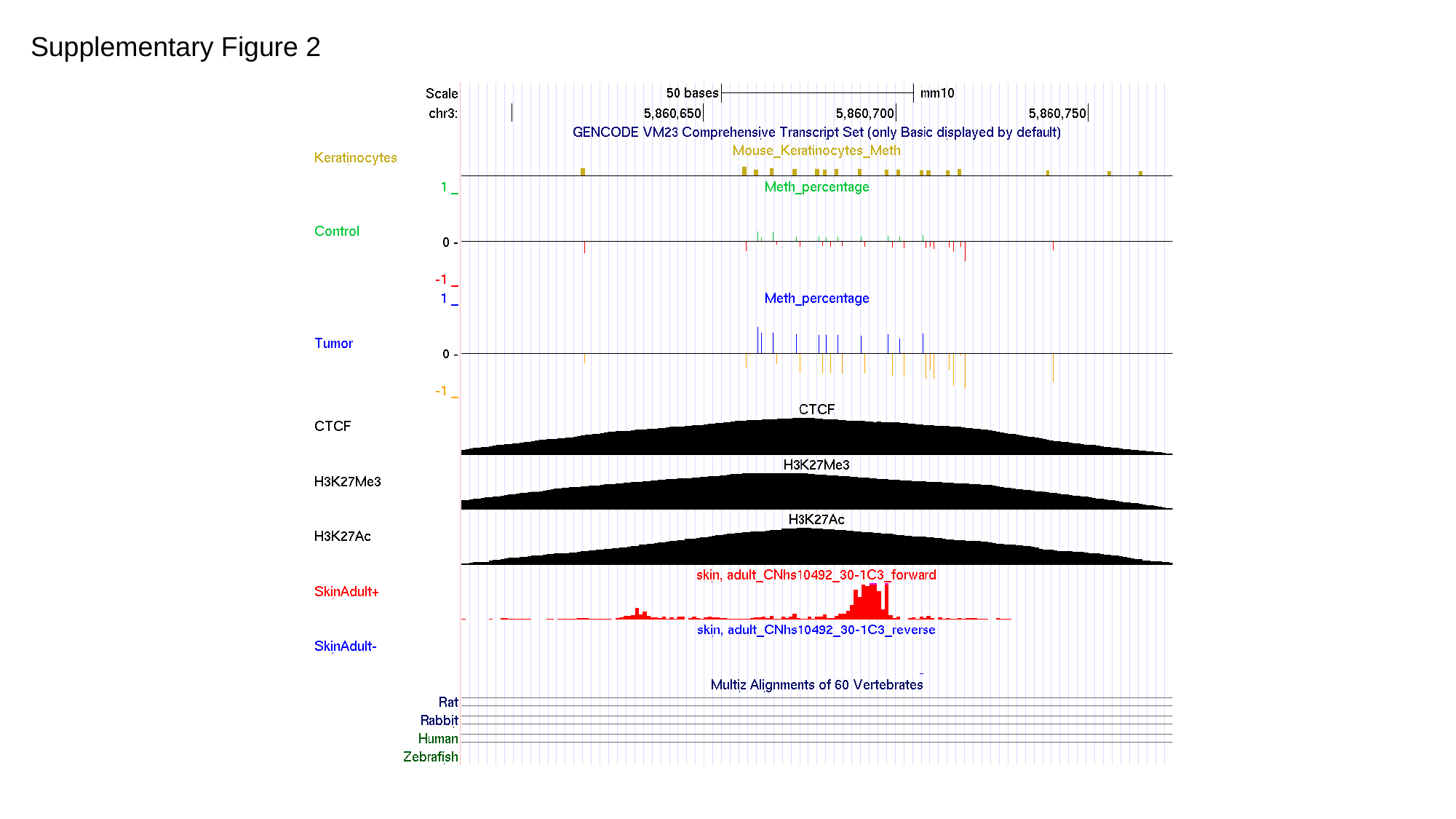

Supplementary Figure 2

## Slide 3
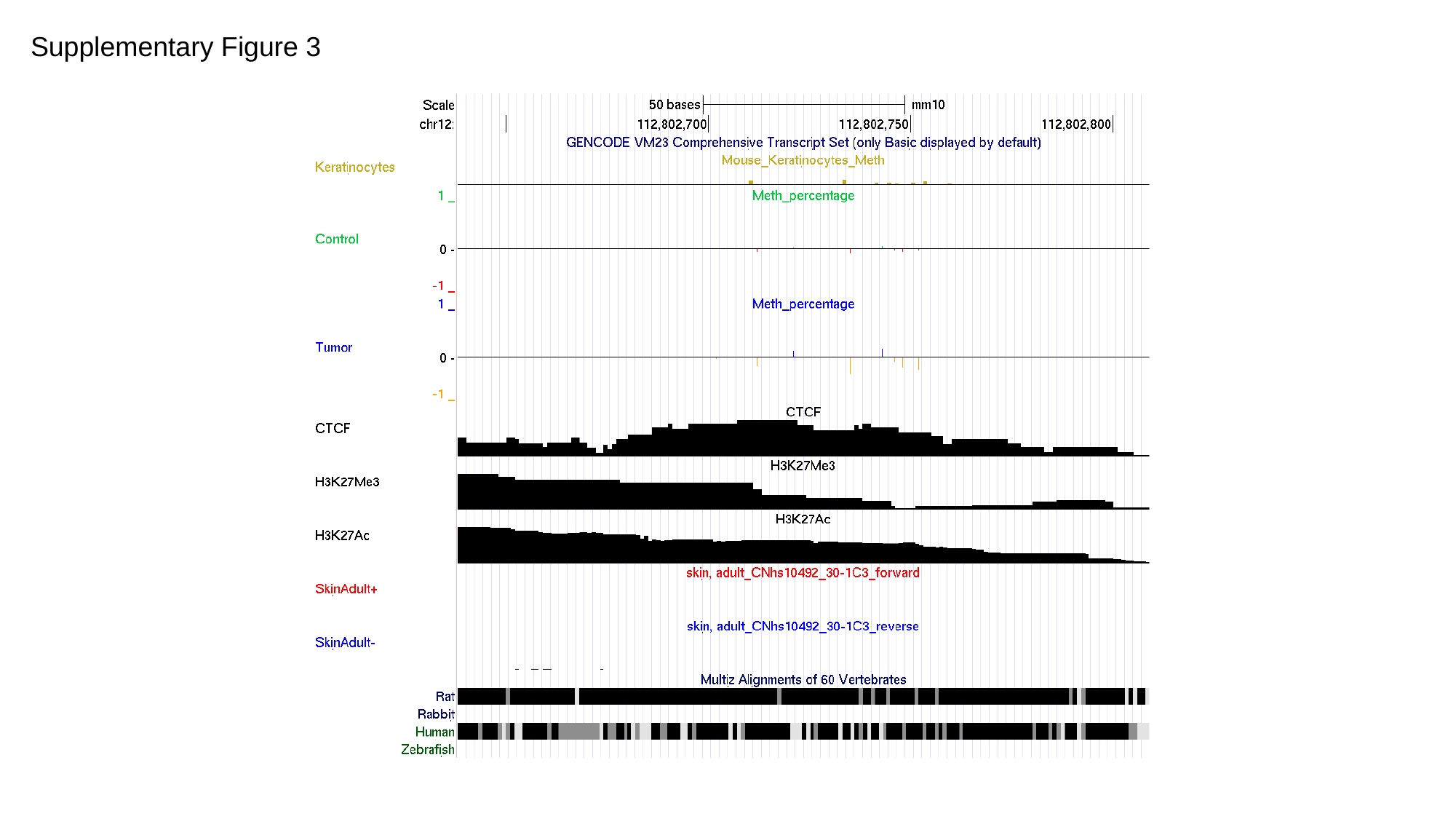

Supplementary Figure 3

## Slide 4
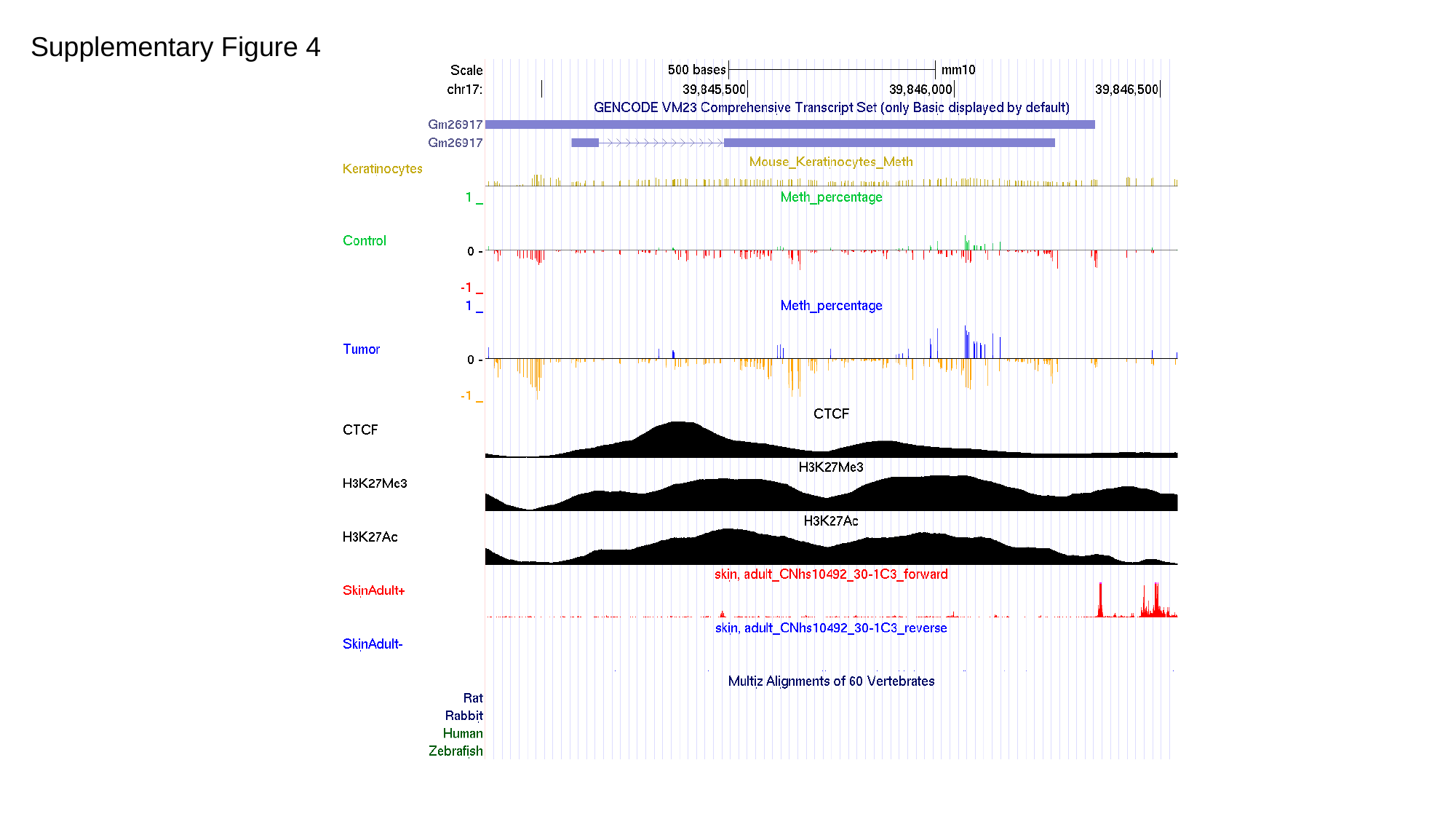

Supplementary Figure 4

## Slide 5
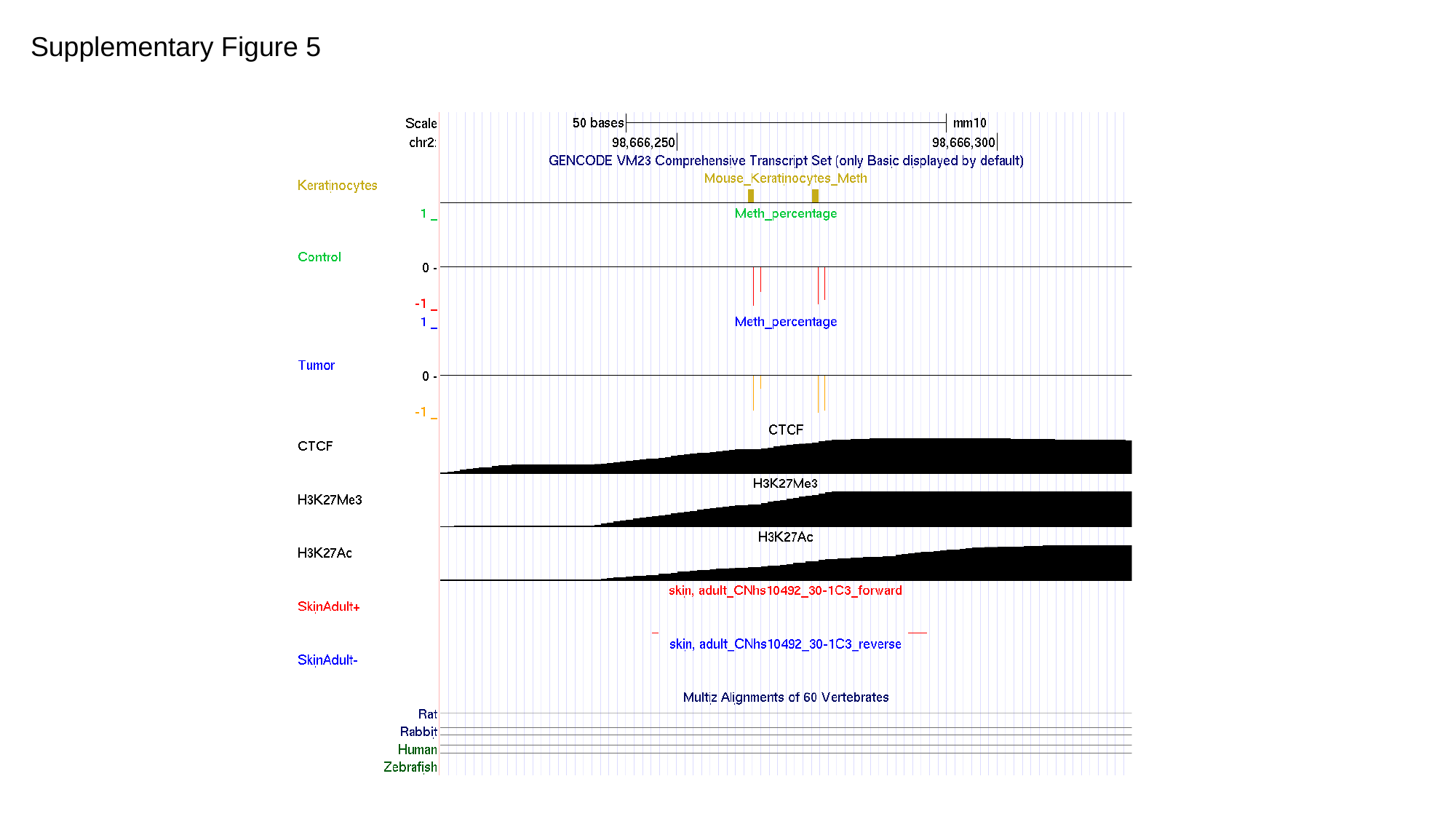

Supplementary Figure 5
